# Perceptions of people with respiratory problems on physician performance evaluation—A qualitative study

**DOI:** 10.1111/hex.12999

**Published:** 2019-11-20

**Authors:** Carolin Sehlbach, Marjan J. B. Govaerts, Sharon Mitchell, Truus G. J. Teunissen, Frank W. J. M. Smeenk, Erik W. Driessen, Gernot G. U. Rohde

**Affiliations:** ^1^ Department of Educational Development and Research Faculty of Health, Medicine and Life Sciences Maastricht University Maastricht The Netherlands; ^2^ School of Health Professions Education Maastricht University Maastricht The Netherlands; ^3^ World Heart Federation Geneva Switzerland; ^4^ Patient Contributor, and Researcher at the Department of Medical Humanities Amsterdam Public Health research institute (APH) Amsterdam UMC Free University Medical Centre Amsterdam The Netherlands; ^5^ Respiratory Medicine Catharina Hospital Eindhoven The Netherlands; ^6^ Department of Respiratory Medicine University Hospital, Goethe University Frankfurt Germany

**Keywords:** empowerment, feedback, lifelong learning, patient and public involvement, patient perspective, performance evaluation, power dynamics, recertification, revalidation, voice

## Abstract

**Background:**

Despite increasing calls for patient and public involvement in health‐care quality improvement, the question of how patient evaluations can contribute to physician learning and performance assessment has received scant attention.

**Objective:**

The objective of this study was to explore, amid calls for patient involvement in quality assurance, patients' perspectives on their role in the evaluation of physician performance and to support physicians’ learning and decision making on professional competence.

**Design:**

A qualitative study based on semi‐structured interviews.

**Setting and Participants:**

The study took place in a secondary care setting in the Netherlands. The authors selected 25 patients from two Dutch hospitals and through the Dutch Lung Foundation, using purposive sampling.

**Methods:**

Data were analysed according to the principles of template analysis, based on an a priori coding framework developed from the literature about patient empowerment, feedback and performance assessment.

**Results:**

The analysis unearthed three predominant patient perspectives: the proactive perspective, the restrained perspective and the outsider perspective. These perspectives differed in terms of perceived power dynamics within the doctor‐patient relationship, patients' perceived ability, and willingness to provide feedback and evaluate their physician's performance. Patients' perspectives thus affected the role patients envisaged for themselves in evaluating physician performance.

**Discussion and conclusion:**

Although not all patients are equally suitable or willing to be involved, patients can play a role in evaluating physician performance and continuing training through formative approaches. To involve patients successfully, it is imperative to distinguish between different patient perspectives and empower patients by ensuring a safe environment for feedback.

## 1 INTRODUCTION

While patient empowerment is gaining momentum,^1^, ^2^ the involvement of patients, hereinafter referred to as patient and public involvement (PPI), in the improvement of health‐care quality, particularly in the evaluation of health‐care professional performance, is often lacking or underreported.^3^, ^4^, ^5^, ^6^, ^7^, ^8^, ^9^ PPI across the medical education continuum ranges from patients' participation in teaching, feedback and assessment or involvement in course design towards partnership and collaboration.^10^ Lalani and colleagues present the worldwide variability of PPI across medical performance processes and call for more collaborative ways of involvement, beyond formal patient feedback and complaints.^11^ Patients are the very essence of why health‐care systems exist and as health‐care consumers, they have a direct stake in the way both quality and providers of care are evaluated.^12^, ^13^

Patients' participation in feedback processes as a form of PPI is generally established through evaluation or satisfaction surveys in which patients communicate their views on care received or evaluate health‐care processes and physicians' professional practice.^4^, ^11^, ^14^ Research findings show that the inclusion of patients' views renders performance evaluations more holistic and transparent, potentially allowing physicians to reflect on their practice.^15^, ^16^, ^17^ Although different in focus from feedback received from peers or other health‐care workers, patient feedback can provide physicians with valuable information on how to improve their learning and performance.^10^ Similarly, patients' evaluations of physician performance can help to make decisions about physician competence and to identify underperforming physicians.^18^ Especially in the assessment of non‐clinical competences, such as communication and professionalism, patient evaluations on physicians' performance can serve as meaningful additional evidence.^11^, ^19^, ^20^, ^21^, ^22^

Patient involvement in physician performance evaluation, however, reaches further than restrictive satisfaction questionnaires. It also entails lay representation in the design of performance evaluation processes or guideline development and strategic planning.^10^ How patient evaluations can contribute to physicians' learning and performance assessment has received little attention—particularly informal patient feedback on the individual functioning of a physician.^23^ Providing feedback is a complex cognitive and affective process and the resulting evaluation is determined by its provider's beliefs, cognition and emotions.^24^ Even though patients' beliefs are often presumed to be known, a recent review on the impact of patient feedback of physician's performance highlights that research on patient feedback from the patient perspective is currently lacking.^25^ Although Lalani et al^11^ disclose that patient characteristics such as age or socio‐economic characteristics may act as barriers to PPI, the authors do not discuss the underlying processes or address patients' perspectives on their role in physician performance evaluation or evaluation systems. In order to understand how to use patient‐generated data, however, we need to explore the assessor perspective and likewise add to a clear conceptual understanding of ‘the patient perspective’.^26^, ^27^ Before we can address such a practical need, we must unpick factors that influence patients' possible role in physicians' learning and performance evaluation, such as their beliefs, preferences and concerns.^10^, ^28^, ^29^ By addressing these gaps, we may be able to achieve a meaningful patient contribution to the evaluation of physician performance processes.^30^, ^31^ The purpose of the present study, therefore, is to answer the question: What are patients' perspectives on their possible role within the evaluation of physician performance and physicians' lifelong learning, particularly in providing feedback?

## 2 METHODS

We conducted a qualitative study based on semi‐structured interviews with the aim to explore patients' notions of evaluation of physician performance, and to better understand their perspectives on their role in the evaluation of physician performance by providing feedback.

### 2.1 Setting and participants

The study was set in The Netherlands that has an obligatory national recertification system in place with limited PPI (Box1).

Box 1 **The Dutch recertification system**

The Dutch recertification system emphasises continuing development over the detection of malpractice. At present, medical specialists must meet the following three requirements after each period of 5 years: (a) they must prove that they have practised medicine sufficiently and regularly (ie ≥ 16 hours per week on average); (b) they must have engaged in continuing medical education (CME) activities worth 200 CME points; and (c) they must have undergone an external quality assessment of their department by a committee of the National Specialty Society. As of 2020, an additional requirement will apply: (d) physicians must demonstrate that they did prepare a personal development plan and participated in an assessment of individual functioning.

We selected patients using purposive sampling based on the following inclusion and exclusion criteria. First, we aimed to include patients who were most likely to have developed a long‐standing or intensive treatment relationship with their physician and who had high levels of experience regarding health‐care delivery. Therefore, we decided to include patients with lung cancer or a chronic lung disease. Second, in order to obtain maximum variation of the patient population, we selected patients with a variation in burden of disease and age. Third, we wanted the sample to reflect varying degrees of patient experiences, views and knowledge, and therefore included not only individual patients, but also patient group members and patient representatives.^32^ Finally, we excluded patients who practised as physicians themselves or who were receiving care from any of the researchers at the time of the study or in the past.

We approached patients in two ways: First, we asked the Dutch Lung Foundation to include a call in their periodical newsletter to their patient panel, inviting interested individuals to contact the first author through the foundation. This resulted in enrolling ten patients. Second, to sample across different diseases within secondary care, we visited the respiratory outpatient clinic of one academic and one non‐academic hospital, which yielded seven and three patients, respectively. To include not only patients, but also their informal carers (mostly partners),^33^ we also enrolled five patient partners, leading to a total number of 25 participants (12 male and 13 female). The mean age of participants enrolled was 65 (ranging from 35 to 82 years old, SD = 10.9).

### 2.2 Data collection

We developed the interview protocol based on literature on patient engagement, evaluation of physician performance and feedback for performance assessment purposes in the health professions. We used the literature^12^, ^14^, ^20^ to include questions that asked explicitly about whether and how patients envisaged a role for themselves in providing informal feedback and evaluating physicians beyond formal satisfaction questionnaires. Having piloted the interview guide (Appendix S1) by conducting the interview with patients who were not included in the study, we revised and simplified the language of the introductory questions. CS interviewed members of the Lung Foundation via phone, while outpatients were interviewed either face‐to‐face after their visit to the clinic or by phone. Semi‐structured interviews lasted 37 minutes on average (SD = 8.1) and were transcribed verbatim. We collected and analysed the data in an iterative process, allowing the analysis to inform subsequent interviews. Data collection and simultaneous analysis took place from June to August 2018, until the research team agreed that thematic saturation was reached.^34^

### 2.3 Patient involvement in this study

Besides enrolling patients as research participants, a patient, TGJT, was also a member of the research team and co‐author. Being a chronic patient herself who has extensively researched the topic of PPI,  TGJT represented the patient voice in the research team by advising on the feasibility and burden of ideas and pointing out potential pitfalls in the study design and conduct. After publication of this study, the results will be distributed in a plain language summary to the research participants and wider patient groups.

### 2.4 Data analysis

We performed a template analysis of our data, which is a form of thematic analysis.^35^ In accordance with this technique, we iteratively applied a sequence of templates to the data set, starting with a priori codes followed by constant modification of themes throughout the analysis. As a first step, the primary researcher CS  familiarized herself with the data and initially coded five interview transcripts based on an a priori coding framework that was developed from the literature about PPI, feedback and performance assessment. A priori codes based on the literature^21^, ^28^, ^36^, ^37^ included perceptions of the doctor‐patient relationship and communication, particularly in light of a potential hierarchical relationship, the role of feedback, and in particular the preferred way of providing feedback, patient empowerment, PPI and patient identity. As a second step, CS modified and replenished the initial codes during the analysis of further interviews, which led to an initial template. This template served to describe whether patients envisaged a role for themselves in the evaluation of physician performance and depicted the levels of trust patients experienced in the relationship with their physician and associated feelings and readiness to provide feedback. As a third step, CS and SM discussed themes; and devised and produced a final template that included themes around patient voices and power dynamics. Based on this final template, CS and FWJMS independently coded and discussed two more transcripts. CS, SM and FWJMS subsequently discussed preliminary interpretations, following which they refined the final template into a focused template (Appendix S2). CS applied this focused template to all interview transcripts and discussed the findings with the entire research team until they reached consensus about the final interpretation.^35^

We ensured validity by conducting a member check among interviewees who confirmed our interpretations. Although all participants were offered the opportunity to participate in the member check, only one participant responded, which had no consequences for data interpretation. We used the software programme ATLAS.ti to manage our data, and the COREQ checklist to report on analysis (Appendix S3).^38^

### 2.5 Reflexivity

In order to maintain the quality of the study, reflective memos were used throughout data collection. CS has a background in health sciences and is a PhD Student in medical education. TGJT conducted scientific research in the area of patient involvement and is also a patient expert and volunteer at the Dutch Lung Foundation. She conjured up the patient's perspective in the study design and analysis. MJBG and EWD are both medical educators. SM is an education manager. Two research team members (FWJMS and GGUR) are respiratory physicians. All members of the research team are involved in medical education, with either a special focus on assessment, continuing education and recertification or patient involvement. Together, the expertise of the research team members contributed to how we incorporated the patient voice into the evaluation of physician performance, on the one hand, while helping us critique its effect on performance evaluation.

## 3 RESULTS

Our analysis led to the construction of three recurring perspectives on the role patients envisaged for themselves in providing feedback and evaluating physicians' performance. In the following sections, we describe which distinct patient perspectives we encountered and how they are characterized.

The predominant perspectives were shaped by patients' personal experiences and the consequences they expected to follow from evaluating their physician. The extent to which patients experienced a power balance within the doctor‐patient relationship seemed to affect the role they envisaged for themselves. The perceived *power dynamics* of the doctor‐patient relationship affected patients' perspective on their *role* in the evaluation of physician performance and their willingness to provide *feedback*. By levels of power, we refer to patients' perceived dependency on their attending physician during treatment. Table 1 summarizes the three predominant perspectives.

**Table 1:** Distinct patient voices in the evaluation of physician performance: what are the predominant perspectives?

	Predominant perspective			
	Proactive perspective	Complacent perspective	Outsider perspective	
			Deliberate	Unintentional
Feedback	Readily provides feedback to improve and safeguard care and prevent harm	Reserved; provides feedback anonymously or indirectly and only when invited	Refrains from providing feedback	Willing to provide interpersonal feedback (when solicited)
Preferred way of communicating feedback	Direct; face‐to‐face	Indirect; written and anonymously	None	Direct when applicable
Power	Power equilibrium; partnership	Power imbalance; hierarchy; respect; compliance	Power imbalance; hierarchy	N/a due to irregular contact
Role	Experienced expert, customer and end user of care	Dependent, vulnerable	Incapable	Incapable, but this might change
Example of quote	‘It is just about the patient’	‘If you are asked to do so, you can. But if not, you think: I will be quiet; otherwise it may not benefit me’	‘Who am I to judge a doctor?’	‘To give a clear answer to your question, ´would you want to judge your respiratory physician?´ No. Not when it concerns competence. But that he is a nice person, yes’

It should be borne in mind that these three overarching, predominant patient perspectives by no means detract from the fact that each patient is unique. Even though we illustrate characteristics of three perspectives, every patient interviewed had their own individual feeling on their perceived ‘place’ to offer feedback, and to decide whether they followed through with this. Some interviewees exhibited characteristics of more than one perspective, or were doubtful of their role in physicians' continuing learning and performance evaluation and shifted between different perspectives. That is, the perspectives presented are not fixed categories but should rather be seen as a continuum across which patients can move, depending on time and context. Regardless of the different perspectives, most interviewees recognized the importance of physicians' continuing learning and performance evaluation systems: ‘continuing training is really important because otherwise you will be overtaken by events at some point’ (Interview 7). Yet, they envisaged their involvement mainly as providing feedback and not as being involved as a lay representative in system design, although some clearly expressed feelings about the need to feel safe within the system. In regard to their role in providing feedback, our interviewees voiced clear ideas about which physician competencies they were able to evaluate. They mostly addressed professionalism and communication as well as collaboration skills.

### 3.1 The proactive perspective

Patients who shared this perspective were assertive and had a relationship with their physician in which they felt power was equally distributed. These patients easily voiced their agreement or dissatisfaction with care received. Considering themselves as health‐care consumers, they felt patients should be at the centre of any care process: ‘I think it is very important that the patient has a voice in this. The patient is ultimately the customer and end user so to say*’* (Interview 15). Consequently, they demanded a say in their treatment and management plan and directly conferred with the attending physician when dissatisfied. By providing constructive feedback, these patients felt they were responsible for and able to customize their own care: ‘It is no longer Mr Consultant in a white coat and we have become more vocal. […] Rather, it is Mr Patient, if you like*’* (Interview 19). Among our interviewees, it was mostly the younger and better‐educated ones who actively engaged in shared decision making and saw themselves as equal partners, although the proactive patient perspective was represented across age groups.

It seemed logical to them that their experienced expert voice should be heard in the evaluation of physician performance. Being part of it was important to them, not for inclusion's sake but for ensuring value and worth within the evaluation process. In particular, they believed their feedback could encourage physicians to reflect, thereby creating a learning opportunity and complementing physicians' self‐assessment: ‘Because a doctor does not know about himself, well some do if they are honest, but they do not always know how they come across to people. And you can only find that out if someone else tells you’ (Interview 9). Pro‐active patients underlined different reasons for engaging in the process of evaluating physicians' performance (Table 1). Although they recognized that their input would probably not have a direct impact on the care they received, they believed it could benefit future patients or the system overall. At the same time, however, these patients comprehended the limitations of their input, realizing that their feedback could only be useful when it concerned specific areas of physician competence that they were actually able to evaluate. Additionally, they believed it should never be a replacement of, but an addition to, peer feedback: ‘I think the feedback and the evaluation of colleagues are also very valuable … they know what they are talking about, but … I think the patient really does belong there as well’ (Interview 2).

### 3.2 The restrained perspective

Patients who showed characteristics of the restrained perspective did not envisage an active role for themselves in the evaluation of physician performance. These patients trusted that the current system would assure physicians' competence and quality of care. Consequently, they were reserved in offering their opinion: ‘Well, that is not necessary. I know that they regulate it from above through, through an organization, or the government… I think that is sufficient’ (Interview 8). Considering their views as subordinate to their physicians, these patients did not spontaneously provide feedback or feel a need to evaluate their physician, especially not when they had complaints because ‘as patient you just do not dare to’ (Interview 8). They fully relied on their physicians' competence and therefore did not venture to question them. As one interviewee explained: ‘Because you also assume so. The doctor is also competent, someone you trust, because if you ask questions, some doctors will ask, wait a minute, do you think I'm not good enough*?*’ (Interview 12). Patients in this group shunned confrontational conversations with their physician, because they were afraid these would negatively affect their relationship, or the treatment received. In the treatment phase, patients felt vulnerable and did ‘not know where [a negative evaluation] would lead to’ (Interview 12). Since they felt uncomfortable criticizing their physician, these patients preferred to give feedback indirectly or anonymously and only when solicited.

Typical of this perspective, moreover, was a perceived power imbalance with senior physicians, whom they portrayed as ‘kings in their realm’ (Interview 12). Noteworthy, these patients experienced less power distance when dealing with younger physicians, GPs, nurses or other health‐care professionals such as physiotherapists. According to them, this power equilibrium was attributed to improved communication and openness, which they directly linked to physicians' age and dedicated skills training: ‘Surely the training is different than in the past, I think they [younger physicians] do learn more communication skills nowadays, I could just talk to them more easily’ (Interview 9). Finally, interviewees in this group doubted that their feedback could promote physicians' learning and, consequently, have any real impact on their performance: ‘I doubt it, I doubt it. … Whether a doctor can do something with it and if (s)he indeed does something with it. I do not know’ (Interview 8). To assure physicians' competence and provision of feedback on quality of care, the restrained patient's voice relied on others within the system: ‘There are people with more energy; they should put their energy into it*’* (Interview 16).

### 3.3 The outsider perspective

Another group of interviewees labelled themselves as outsider: ‘I consider myself too much of an outsider to be asked to evaluate my doctor's performance*’* (Interview 7). Within this predominant perspective, we distinguished between the unintentional and the deliberate outsiders, both of whom felt unable to evaluate physicians' performance: ‘Who am I to evaluate a doctor?’ (Interview 15). Both groups doubted whether their opinion could contribute to the continuing development of their physician, albeit for different reasons.

Patients holding the unintentional outsider perspective pointed to generic problems in the health‐care system, such as the brevity and irregularity of encounters caused by tight schedules and ever‐changing physicians. These patients might be more ready to provide feedback if they had more regular and direct contact with their specialist. Even though patients with this perspective would occasionally give direct feedback when dissatisfied, they were hesitant to judge their physician's competence because they hardly knew their physician: ‘I think you should have seen such a doctor a couple of times, before you are able to give an assessment’ (Interview 4).

For patients showing characteristics of the deliberate outsider perspective, on the other hand, power imbalance played a more prominent role. Being more susceptible to power dynamics, they automatically considered their attending physicians as the expert possessing the necessary skills and knowledge, and thus as superior within the context of the relationship, thereby putting their full trust in them. More specifically, patients sharing this perspective felt they lacked insight and knowledge in that field and felt unable to evaluate their physician: ‘Really, on the performance of a doctor, who am I to give a judgement on that?’ (Interview 7). Unlike the restrained perspective, patients relating to the deliberate outsider perspective truly felt incompetent to judge physicians and therefore preferred not to give feedback, not even when anonymous or solicited.

## 4 DISCUSSION

With this study, we aimed to explore patients' perspectives on their role within the evaluation of physician performance. We were able to define three predominant patient perspectives that depended on the extent to which patients felt competent to take this role and to which they experienced a power balance within the doctor‐patient relationship: the proactive perspective, the restrained perspective and the outsider perspective.

Reflecting on the challenges inherent in PPI,^31^, ^39^ our results underline that there is no such thing as a ‘collective’ patient voice, but that a multitude of patient perspectives must be considered. Indeed, not only are patient perspectives individually bound, they are tied to a specific moment in time.^26^, ^29^, ^31^ Patients might change their perspective depending on context (e.g. dependency on physician due to disease status and number of contacts with physician) and therefore cannot be pegged into a fixed category. Examining the conceptualization of ‘the patient perspective’, Rowland, McMillan, McGillicuddy, Richards 26 pointed out that patient perspectives are temporal, contextual and based on embodied knowledge and experiences of vulnerability. This observation ties in with our finding that perceived power dynamics appear to influence patients' readiness to play a role in the evaluation of physician performance. That is, the extent to which they experienced a power (im)balance within the doctor‐patient relationship seemed to have a direct impact on their voicing behaviour. Feelings of vulnerability and dependency during treatment impacted negatively on the extent to which interviewees felt able and/or willing to evaluate physicians' competence.^37^ This aligns with previous definitions of ‘power’ in social interactions in health care, characterized by an often unequal relationship between physicians and patients, in which patients are vulnerable and have to rely on and trust the medical experts.^40^ Others, however, assign power and autonomy to patients instead, describing them as health‐care consumers and physicians as those being vulnerable, for instance when fearing that patient feedback may be defamatory and cause reputational damage.^36^, ^41^ Conceptualizations of patients being autonomous and powerful health‐care consumers align with the proactive patient perspective in our study where patients found themselves in power equilibrium with their physicians.^39^

Similarly, Tazzyman et al^42^ describe how power dynamics may affect acceptance of patient feedback by physicians. Their study findings illustrate how medical specialists struggle accepting or oppose patient feedback and link this to historical power difference and hierarchy as well as a lack of common language between patients and physicians.^42^ The latter argument, however, might be invalid for patient experts or representatives, who are well trained to discuss patient perspectives with professionals and policy makers.^32^ Consequently, physicians might more readily accept feedback from these patients experts, presuming they have an understanding of their medical work.^43^ This argues for more effort in the field of patient education and improved power dynamics, and suggests a change in the future once reliable patient‐generated information become increasingly available.

Altogether, this highlights that not only the provision of feedback, but also its acceptance can be challenging for patients and physicians, respectively. The type of feedback, its credibility and the competence addressed, determines whether physicians accept patient feedback.^43^ Physicians might accept patient feedback on their communication more easily, whereas they might consider for instance feedback on medical expertise as not credible.^44^, ^45^, ^46^ This is very much in line with our results, which show that, although patients felt they could evaluate physicians' communication or professionalism, they relied on other health‐care professionals to evaluate physicians' medical expertise.

Likewise, patient evaluations can be combined with other performance evaluations, particularly for non‐medical competencies, as suggested by our participants and supported by previous research.^47^ This combination can be useful for formative purposes to induce physicians' reflection and insights into the strengths and weaknesses of their professional practice. It holds particularly true, however, for summative processes such as recertification elsewhere coined ‘revalidation’ or ‘maintenance of certification’. Countries use recertification systems, to improve processes and outcomes of patient care, while ensuring patient safety. Based on standards for physicians' competence and fitness to practice, these systems aim to prevent and concurrently detect malpractice. Alongside regulatory approaches, most systems employ an educational approach to support physicians' continuing professional development and lifelong learning.^15^ Patients can be involved in recertification through providing feedback to their physician. The revalidation system in the United Kingdom for instance already structurally includes patient feedback in regulatory processes.^48^, ^49^, ^50^

### 4.1 Implications for clinicians and policymakers

Some patients can provide feedback on processes and outcomes of care, aiding quality improvement. Formal patient feedback on service delivery can for instance induce physicians' learning and likewise improve the care delivered.^51^ It is, however, paramount to not only ask patients to provide feedback on care received but to invite patients to collaborate with policymakers and medical content experts on quality guidelines or new implementations.^52^

Our results fully support the need to design feedback systems that cater to patients' diversity and unique contributions. It remains a boundary condition, however, to collect numeric and narrative feedback from multiple patients and through credible formats.^25^, ^53^, ^54^ Patients suggested written forms to be compact, straightforward and easy to understand. Most patients interviewed preferred anonymous forms or face‐to‐face discussions mediated by a third party. Offering paper‐based or electronic questionnaires might for instance help to include various perspectives from heterogeneous patient populations, including patients who would otherwise refrain from providing feedback themselves, such as patients showing characteristics of the restrained or the outsider perspective, or those who would be left out based on their socio‐economic status, age or ethnicity.^37^ For instance, by offering the opportunity to provide feedback anonymously and in a neutral environment outside the doctor‐patient relationship, thereby creating a ‘safe space’, we may encourage restrained patients to become involved in physicians' learning. In addition, we must channel efforts into achieving better power dynamics, trust and prolonged relationships in health care so that outsiders can become insiders, if desired. This, however, requires paying attention to training physicians in asking patients for informal feedback in ways that are non‐threatening. It further implies organizing health care in a way that patients and physicians can establish trusting relationships, through continuity of care and increased patient education.

### 4.2 Strengths and weaknesses

First, the main strength of our work is the rigour with which we performed the data analysis, characterized by the iterative analysis process. The reflexivity and the deliberation within a mixed research team form an additional strength. Second, we sampled purposefully across a range of, mostly chronic, lung diseases, age and educational background in order to present a heterogeneous patient group. Patients with chronic disease can be considered experts regarding their health or disease status, treatment and health‐care service received and may likely perceive the doctor‐patient relationship differently than patients with acute diseases do. This research can help physicians to become aware of predominant perspectives among their own patients and consult them accordingly. Altogether, our results enable us to suggest policy implications regarding patient participation in the organization of health care, based on the patient perspectives we explored.

Some potential limitations of the present research are worth considering. First, we only included patients who resided in the Netherlands. As patient perspectives are context‐bound, replications of this research in another country with a different health‐care system, diverse cultural context and performance evaluation system may produce different results. This process may yield different results depending on the context of the study as well as the patients interviewed. Our findings, however, remain relevant for a wider audience and give direction for future research. Second, patients reflected on their future role in the evaluation of physician performance, potentially without having actual experience having provided it at least in the way they proposed. Third, we only included patients with lung cancer or a chronic lung disease who, moreover, tend to be older adults. Younger patients, other disease areas or people with non‐chronic diseases may have perceived their role in performance evaluation differently. Fourth, a number of participants were self‐selected volunteers from the Dutch Lung Foundation's patient panel. This self‐selected group may have been more vocal than their outpatient peers whom we approached individually, since they already played an active role in giving their opinion and feedback on the health‐care system. The volunteers lived across the country and were often limited in travel due to their medical condition, which required us to conduct interviews by telephone. Finally, the authors' backgrounds have most certainly shaped their view on the topic of patient involvement in the evaluation of physician performance. It required constant deliberation within the team, and reminders of being critical and open towards any interviewees' statements, which was facilitated by TGJT as patient expert.

## 5 CONCLUSION

Patients have different perspectives on their roles in the evaluation of physician performance. This research suggests that, to be able to support physicians' learning and improve care, we must first gain a better understanding of patients' perspectives and reconceive ‘the patient’ within health care.^32^, ^39^ Our findings highlight the ethical and moral obligation to acknowledge the unique contribution of individual patient voices. As not every patient is equally suitable or wishes to evaluate care processes or to provide feedback on physician performance, we must strive for the correct balance between patient empowerment and respect for patients' unique perspectives.^37^, ^55^ This research importantly underlines the need to equalize the perceived power balance in the doctor‐patient relationship and to invite patients to evaluate physician performance in line with their individual preferences. Ultimately, in an era of more complex and demanding health‐care systems, there is growing need to work with patients to design, implement and improve the evaluation of physician performance.

## CONFLICT OF INTEREST

The authors declare no competing interests other than the funding listed above. Moreover, all authors have completed the Unified Competing Interest form (available on request from the corresponding author) and declare: no support from any organization for the submitted work other than reported; no financial relationships with any organizations that might have an interest in the submitted work in the previous three years; and no other relationships or activities that could appear to have influenced the submitted work.

## **AUTHORS**'** CONTRIBUTIONS**

All authors designed and conceived the study. CS wrote the research plan, collected and analysed the data and drafted and revised the paper. She is guarantor. MJBG analysed the data, and drafted and revised the paper. SM drafted and revised the paper. TGJT analysed the data, and drafted and revised the paper. FWJMS analysed the data, and drafted and revised the paper. ED drafted and revised the paper. GGUR drafted and revised the paper. All authors engaged in the final steps of analysing the data. As corresponding author, [first author] attests that all listed authors meet authorship criteria and that no others meeting the criteria have been omitted.

## ETHICAL APPROVAL

We informed potential study participants about the research as soon as they contacted the Dutch Lung Foundation or registered for their consultation at the outpatient clinic. We emphasized that the research was entirely independent of the care received and that the treating physician would not receive any information about the interview. All participants gave written and oral informed consent.

We obtained ethics approval from both the Netherlands Association for Medical Education (NVMO: file number 1031) and the ethics committees of the two participating hospitals (MEC [location]: file number 2018‐0479; and MEC‐[location]: file number nWMO‐2018.56).

## TRANSPARENCY STATEMENT

The lead author and guarantor of this manuscript affirms that this manuscript is an honest, accurate and transparent account of the study being reported; that no important aspects of the study have been omitted; and that any discrepancies from the study as originally planned have been explained.

## Data Availability

The data that support the findings of this study are available on request from the corresponding author. The data are not publicly available due to privacy or ethical restrictions.

## References

[1] Chu LF , Utengen A , Kadry B , et al. “Nothing about us without us”—patient partnership in medical conferences. BMJ. 2016;354:i3883 27628427 10.1136/bmj.i3883

[2] Richards T , Montori VM , Godlee F , Lapsley P , Paul D . Let the patient revolution begin. BMJ. 2013;346:f2614. 23674136 10.1136/bmj.f2614

[3] Armstrong N , Herbert G , Aveling EL , Dixon‐Woods M , Martin G . Optimizing patient involvement in quality improvement. Health Expect. 2013;16(3):e36‐e47. 23374430 10.1111/hex.12039 PMC3883095

[4] Groene O , Lombarts MJMH , Klazinga N , Alonso J , Thompson A , Suñol R . Is patient‐centredness in European hospitals related to existing quality improvement strategies? Analysis of a cross‐sectional survey (MARQuIS study). Qual Saf Health Care. 2009;18(Suppl 1):i44‐i50. 19188461 10.1136/qshc.2008.029397 PMC2629879

[5] Price A , Schroter S , Snow R , et al. Frequency of reporting on patient and public involvement (PPI) in research studies published in a general medical journal: a descriptive study. BMJ Open. 2018;8(3):e020452 10.1136/bmjopen-2017-020452 PMC5875637 29572398

[6] Richards T , Coulter A , Wicks P . Time to deliver patient centred care. BMJ. 2015;350:h530. 25670197 10.1136/bmj.h530

[7] Liabo K , Boddy K , Burchmore H , Cockcroft E , Britten N . Clarifying the roles of patients in research. BMJ. 2018;361:k1463. 29636343 10.1136/bmj.k1463

[8] Staniszewska S , Brett J , Simera I , et al. GRIPP2 reporting checklists: tools to improve reporting of patient and public involvement in research. BMJ. 2017;358:j3453. 28768629 10.1136/bmj.j3453 PMC5539518

[9] Boivin A , L'Esperance A , Gauvin FP , et al. Patient and public engagement in research and health system decision making: a systematic review of evaluation tools. Health Expect. 2018;21(6):1075‐1084. 30062858 10.1111/hex.12804 PMC6250878

[10] Regan de Bere S , Nunn S . Towards a pedagogy for patient and public involvement in medical education. Med Educ. 2016;50(1):79‐92. 26695468 10.1111/medu.12880

[11] Lalani M , Baines R , Bryce M , et al. Patient and public involvement in medical performance processes: a systematic review. Health Expect. 2019;22(2):149‐161. 30548359 10.1111/hex.12852 PMC6433319

[12] Dent M , Pahor M . Patient involvement in Europe – a comparative framework. J Health Organ Manag. 2015;29(5):546‐555. 26222875 10.1108/JHOM-05-2015-0078

[13] The DA , Lecture L . Quality assurance in health care: consumers' role. Qual Saf Health Care. 1992;1(4):247‐251. 10.1136/qshc.1.4.247 PMC1055035 10136873

[14] Campbell JL , Richards SH , Dickens A , Greco M , Narayanan A , Brearley S . Assessing the professional performance of UK doctors: an evaluation of the utility of the general medical council patient and colleague questionnaires. Qual Saf Health Care. 2008;17(3):187‐193. 18519625 10.1136/qshc.2007.024679

[15] Archer J , Regan de Bere S , Nunn S , Clark J , Corrigan O . “No one has yet properly articulated what we are trying to achieve”: a discourse analysis of interviews with revalidation policy leaders in the United Kingdom. Acad Med. 2015;90(1):88‐93. 25229194 10.1097/ACM.0000000000000464

[16] Brennan TA , Horwitz RI , Duffy F , Cassel CK , Goode LD , Lipner RS . The role of physician specialty board certification status in the quality movement. JAMA. 2004;292(9):1038‐1043. 15339894 10.1001/jama.292.9.1038

[17] Walshe K , Benson L . Time for radical reform. BMJ. 2005;330(7506):1504‐1506. 15976427 10.1136/bmj.330.7506.1504 PMC558466

[18] Baker R , Smith A , Tarrant C , McKinley RK , Taub N . Patient feedback in revalidation: an exploratory study using the consultation satisfaction questionnaire. Br J Gen Pract. 2011;61(591):e638‐e644. 22152843 10.3399/bjgp11X601343 PMC3177132

[19] Moonen‐van Loon JM , Overeem K , Govaerts MJ , Verhoeven BH , van der Vleuten CP , Driessen EW . The reliability of multisource feedback in competency‐based assessment programs: the effects of multiple occasions and assessor groups. Acad Med. 2015;90(8):1093‐1099. 25993283 10.1097/ACM.0000000000000763

[20] Kuper A , Reeves S , Albert M , Hodges BD . Assessment: do we need to broaden our methodological horizons? Med Educ. 2007;41(12):1121‐1123. 18045364 10.1111/j.1365-2923.2007.02945.x

[21] Ha JF , Longnecker N . Doctor‐patient communication: a review. Ochsner J. 2010;10(1):38‐43. 21603354 PMC3096184

[22] Little P , Everitt H , Williamson I , et al. Observational study of effect of patient centredness and positive approach on outcomes of general practice consultations. BMJ. 2001;323(7318):908‐911. 11668137 10.1136/bmj.323.7318.908 PMC58543

[23] Evans RG , Edwards A , Evans S , Elwyn B , Elwyn G . Assessing the practising physician using patient surveys: a systematic review of instruments and feedback methods. Fam Pract. 2007;24(2):117‐127. 17264071 10.1093/fampra/cml072

[24] Kogan JR , Conforti LN , Bernabeo EC , Durning SJ , Hauer KE , Holmboe ES . Faculty staff perceptions of feedback to residents after direct observation of clinical skills. Med Educ. 2012;46(2):201‐215. 22239334 10.1111/j.1365-2923.2011.04137.x

[25] Baines R , Regan de Bere S , Stevens S , et al. The impact of patient feedback on the medical performance of qualified doctors: a systematic review. BMC Med Educ. 2018;18(1):173. 30064413 10.1186/s12909-018-1277-0 PMC6069829

[26] Rowland P , McMillan S , McGillicuddy P , Richards J . What is "the patient perspective" in patient engagement programs? Implicit logics and parallels to feminist theories. Health (London). 2017;21(1):76‐92. 27130651 10.1177/1363459316644494

[27] Velpry L . The patient's view: issues of theory and practice. Cult Med Psychiatry. 2008;32(2):238‐258. 18299968 10.1007/s11013-008-9086-2

[28] Sheldon H , Swain D , Harriss L The patient voice in revalidation: a discourse analysis Oxford. Oxford, UK: Picker Institute Europe; 2011.

[29] Rose D . Patient and public involvement in health research: ethical imperative and/or radical challenge? J Health Psychol. 2014;19(1):149‐158. 24058120 10.1177/1359105313500249

[30] Price DW , Biernacki H , Nora LM . Can maintenance of certification work? Associations of MOC and improvements in physicians' knowledge and practice. Acad Med. 2018;93(12):1872‐1881;Publish Ahead of Print. 29952770 10.1097/ACM.0000000000002338

[31] Tritter JQ . Revolution or evolution: the challenges of conceptualizing patient and public involvement in a consumerist world. Health Expect. 2009;12(3):275‐287. 19754691 10.1111/j.1369-7625.2009.00564.x PMC5060496

[32] Williamson C . How do we find the right patients to consult? Qual Prim Care. 2007;15:195‐199.

[33] BMJ . What does the BMJ mean by patient involvement and co‐production? Education. https://www.bmj.com/sites/default/files/attachments/resources/2017/03/guidancepatientinvolvement.pdf. Accessed May 23, 2018.

[34] Hennink MM , Kaiser BN , Marconi VC . Code saturation versus meaning saturation: how many interviews are enough? Qual Health Res. 2017;27(4):591‐608. 27670770 10.1177/1049732316665344 PMC9359070

[35] King N . Doing template analysis In: Symon G, Cassell C, eds. Qualitative organizational research: core methods and current challenges. London, UK: Sage; 2012.

[36] Goodyear‐Smith F , Buetow S . Power issues in the doctor‐patient relationship. Health Care Anal. 2001;9(4):449‐462. 11874258 10.1023/A:1013812802937

[37] Ocloo J , Matthews R . From tokenism to empowerment: progressing patient and public involvement in healthcare improvement. BMJ Qual Saf. 2016;25(8):626‐632. 10.1136/bmjqs-2015-004839 PMC4975844 26993640

[38] Tong A , Sainsbury P , Craig J . Consolidated criteria for reporting qualitative research (COREQ): a 32‐item checklist for interviews and focus groups. Int J Qual Health Care. 2007;19(6):349‐357. 17872937 10.1093/intqhc/mzm042

[39] Rowland P , Kumagai AK . Dilemmas of representation: patient engagement in health professions education. Acad Med. 2018;93(6):869‐873. 29068822 10.1097/ACM.0000000000001971

[40] Bending ZJ . Reconceptualising the doctor‐patient relationship: recognising the role of trust in contemporary health care. J Bioeth Inq. 2015;12(2):189‐202. 25124984 10.1007/s11673-014-9570-z

[41] Fochsen G , Deshpande K , Thorson A . power imbalance and consumerism in the doctor‐patient relationship: health care providers' experiences of patient encounters in a rural district in India. Qual Health Res. 2006;16(9):1236‐1251. 17038755 10.1177/1049732306293776

[42] Tazzyman A , Ferguson J , Hillier C , et al. The implementation of medical revalidation: an assessment using normalisation process theory. BMC Health Serv Res. 2017;17(1):749. 29157254 10.1186/s12913-017-2710-5 PMC5697083

[43] Ferguson J , Wakeling J , Bowie P . Factors influencing the effectiveness of multisource feedback in improving the professional practice of medical doctors: a systematic review. BMC Med Educ. 2014;14(1):76. 24725268 10.1186/1472-6920-14-76 PMC4011765

[44] DiMatteo MR , DiNicola DD . Sources of assessment of physician performance: a study of comparative reliability and patterns of intercorrelation. Med care. 1981;829‐842. 7278418 10.1097/00005650-198108000-00003

[45] Murray‐García JL , Selby JV , Schmittdiel J , Grumbach K , Quesenberry CPJ . Racial and ethnic differences in a patient survey: patients' values, ratings, and reports regarding physician primary care performance in a large health maintenance organization. Med Care. 2000;38(3):300‐310. 10718355 10.1097/00005650-200003000-00007

[46] Asprey A , Campbell JL , Newbould J , et al. Challenges to the credibility of patient feedback in primary healthcare settings: a qualitative study. Br J Gen Pract. 2013;63(608):e200‐e208. 23561787 10.3399/bjgp13X664252 PMC3582979

[47] Hill JJ , Asprey A , Richards SH , Campbell JL . Multisource feedback questionnaires in appraisal and for revalidation: a qualitative study in UK general practice. Br J Gen Pract. 2012;62(598):e314‐e321. 22546590 10.3399/bjgp12X641429 PMC3338052

[48] General Medical Council . Guidance on supporting information for appraisal and revalidation. 2018; http://www.gmc-uk.org/-/media/documents/rt%2013-supporting-information-for-appraisal-and-revalidation%2013-dc5485_pdf-55024594.pdf. Accessed March 20, 2019.

[49] Merkur S , Mossialos E , Long M , McKee M . Physician revalidation in Europe. Clin Med (Lond). 2008;8(4):371‐376. 18724601 10.7861/clinmedicine.8-4-371 PMC4952927

[50] Sehlbach C , Govaerts MJ , Mitchell S , Rohde GGU , Smeenk FWJM , Driessen EW . Doctors on the move: a European case study on the key characteristics of national recertification systems. BMJ Open. 2018;8(4):e019963. 10.1136/bmjopen-2017-019963 PMC5905769 29666131

[51] Marsh C , Peacock R , Sheard L , Hughes L , Lawton R . Patient experience feedback in UK hospitals: What types are available and what are their potential roles in quality improvement (QI)? Health Expect. 2019;22(3):317‐326. 31016863 10.1111/hex.12885 PMC6543142

[52] Kitto S , Bell M , Peller J , et al. Positioning continuing education: boundaries and intersections between the domains continuing education, knowledge translation, patient safety and quality improvement. Adv Health Sci Educ Theory Pract. 2013;18(1):141‐156. 22167577 10.1007/s10459-011-9340-1

[53] Overeem K , Lombarts MJMH , Arah OA , Klazinga NS , Grol RPTM , Wollersheim HC . Three methods of multi‐source feedback compared: a plea for narrative comments and coworkers' perspectives. Med Teach. 2010;32(2):141‐147. 20163230 10.3109/01421590903144128

[54] Donnon T , Al Ansari A , Al Alawi S , Violato C . The reliability, validity, and feasibility of multisource feedback physician assessment: a systematic review. Acad Med. 2014;89(3):511‐516. 24448051 10.1097/ACM.0000000000000147

[55] Lyons M . Should patients have a role in patient safety? a safety engineering view. Qual Saf Health Care. 2007;16(2):140‐142. 17403763 10.1136/qshc.2006.018861 PMC2653153

