# Supplementary material for: Perceptions of people with respiratory problems on physician performance evaluation—A qualitative study
Source: Health Expect. 2019 Nov 20;23(1):247–55. doi: 10.1111/hex.12999 (PMC6978864; doi:10.1111/hex.12999)
Supplement: Supplementary file 1 [file HEX-23-247-s001.docx]

**Appendix I: Interview protocol**

**Project:** Patients’ role in evaluation of physician performance

**Time of interview:**

**Date and place:**

**Interviewer:**

**Interviewee:**

**Position of the Interviewee:**

(*study aim*) Thank you for taking the time today to talk to me about your potential role as a patient in evaluation of physician performance. As described in the information letter, I would like to understand patients’ role in performance evaluation better. I am specifically interested in how important you think evaluation of physician performance is, whether you as a patient envisage a role for yourself in physician evaluation and what this role could look like. It is not my intention to judge you, nor your physician. There are no right or wrong answers. I am primarily interested in your opinion.

(*procedure*) Please feel free to tell me how you think about it. We will start with some general questions about evaluation of physician performance and its importance to you, and then go into your role within the system. You do not need to tell me the name of your physician, as this research concerns the role of patients in evaluation of physician performance in general. I will audio record the interview so that I can concentrate on what you say. It will also enable me to listen back to it later and to transcribe it.

(*anonymity*) As described in the information letter, all the information and data concerning you, including everything you tell me, will remain strictly confidential. Moreover, as we will replace your name by a code, you will remain anonymous.

Do you have any questions left that the information sheet did not answer?

If you agree, I will now start the recording. We can stop the recording anytime you wish.

Opening:

For this research, we are interested in your opinion as a patient on how we can encourage pulmonologists to continue to learn how to provide good care, and what role you can play in this.

Introductory questions:

- How would you describe a ‘good’ respiratory specialist?
- Have you been asked before to give your opinion on your respiratory specialists? How? With which aim? How did you experience that?
  - You could compare it with a satisfaction survey in an online store or your supermarket.
- Do you have an idea about how doctors can continue to learn? How can they demonstrate that they provide good care? Do you know that revalidation aimed to promote lifelong learning by medical specialists exists? Do you know what it entails?
- Doctors must always continue to learn and develop. There are so many new developments that they must keep abreast of. In the Netherlands, respiratory specialists (and other medical specialists) must demonstrate every 5 years that they have continued to develop themselves, so that they are equipped with up-to-date knowledge and skills to deliver good care. We call this revalidation.
- What do you think of this? Which important points do you feel physicians could or should learn? If you could give advice, what would you recommend?

Core questions:

- Would you consider it important for doctors to undergo re-registration?
  - If so, why?

*Providing feedback to support the learning process of doctors*

Doctors receive information about their functioning in a variety of ways, from which they can learn. How would you feel if you were asked to give your opinion as a patient about the performance of your doctor? ... (the level of knowledge, the way of talking, cooperation, etc.)

- Would you like to be asked? Why? What could this look like?
- What do you expect from your doctor?

*Assessing how to support the learning process of doctors*

- How would you like it if you were asked to evaluate your physician?
- Do you think you could play a role in your physician’s evaluation?
  - What would this role look like?
  - Which role could you play? Why?
  - Which role would you want to play? Why?
  - What would you expect your physician to learn from that?
- Would you like to discuss this with your attending physician?

Final question:

- Are there any other aspects that you would like to discuss or share?

Thank you for participating. I have a small token of appreciation for you.

**Note:** For purposes of enhanced coherence and legibility, quotes and interview protocol have been subject to light editing before journal submission. The authors, however, based their analysis on the original, untidied-up transcriptions and protocol.
