# Supplementary material for: Perceptions of people with respiratory problems on physician performance evaluation—A qualitative study
Source: Health Expect. 2019 Nov 20;23(1):247–55. doi: 10.1111/hex.12999 (PMC6978864; doi:10.1111/hex.12999)
Supplement: Supplementary file 2 [file HEX-23-247-s002.docx]

Appendix II

Simplified version of final template for data analysis

1. Patient perspective
   1. Complacent
   2. Pro-active
   3. Outsider
      1. Deliberate
      2. Unintentional
2. Feedback
   1. Preferred way of communicating feedback
      1. Anonymous
   2. Reasons to provide feedback
      1. For physician’s insight and reflection
      2. Complaint
3. Doctor-patient relationship
   1. Hierarchy
   2. Role patient
      1. Capability
